# The Implicit Relational Assessment Procedure as a Measure of Sexual Orientation in Heterosexual, Bisexual, and Lesbian/Gay Men and Women

**DOI:** 10.1007/s10508-025-03241-z

**Published:** 2025-11-10

**Authors:** Liadh Timmins, Dermot Barnes-Holmes, Qazi Rahman

**Affiliations:** 1 Faculty of Medicine, Health and Life Science, School of Psychology, Swansea University, Vivian Tower, Singleton Park, Swansea, SA2 8PP Wales, UK; 2 Department of Psychology, Institute of Psychiatry, Psychology and Neuroscience, King’s College London, London, UK; 3 School of Psychology, Ulster University, Coleraine, Northern Ireland

**Keywords:** Sexual attraction, Sexual orientation, Implicit Relational Assessment Procedure, Bisexuality

## Abstract

Previous research has suggested that scores generated using a sexual orientation Implicit Relational Assessment Procedure (IRAP) correlate with self-reported sexual orientation in gay and heterosexual men and have a strong ability to differentiate the two groups (Timmins et al., 2016). The present study sought to replicate this work and to determine how a range of groups that differ in terms of self-reported sexual orientation perform on the measure. Lesbian/gay, heterosexual, and bisexual women (*n* = 99) and men (*n* = 96) completed a sexual orientation IRAP and self-report measures of sexual attraction and behavior. The IRAP involved responding “True” or “False” to pictures of nude males and females paired with words meaning attractive or unattractive. Participants were required to respond as if all men were attractive and all women were unattractive for half of the IRAP’s test blocks, and vice versa for the other half. Response latencies were recorded, and *D*-IRAP scores were calculated for overall responses, female responses, and male responses. Significant correlations were found between all *D*-IRAP scores and all corresponding self-report measures for men (*r*s = .38–.64). This was also true for women (*r*s = .24–.53) with a single exception: the *D*-IRAP score for responses to men with self-reported sexual attraction to men. Similarly, almost all *D*-IRAP scores significantly differentiated sexual orientation groups (areas under the curve = .64–.92), apart from bisexual and lesbian/gay women. These findings suggest the IRAP is a useful ex situ measure of sexual orientation.

**Supplementary Information:**

The online version contains supplementary material available at 10.1007/s10508-025-03241-z.

## Introduction

Sexual orientation generally refers to one’s relative sexual attraction to men, women, or both (Bailey et al., 2016). Sexual orientation and its various aspects can be quantified with a variety of measures. Most research concerned with this construct uses self-reports of in situ experiences, with gold-standard applications of this technique requiring participants to disclose their identity, attraction, and behavior (Badgett et al., 2009). While this type of measure is sufficient for most purposes, a range of ex situ measures have also been deployed, including measures of genital arousal (Chivers et al., 2010), pupil dilation (Rieger & Savin-Williams, 2012), viewing time (Lippa, 2017), eye-tracking (Wenzlaff et al., 2016), and various “implicit” measures (Snowden et al., 2008; Timmins et al., 2016). Although researchers often conceive of these as different measures or facets of the same overarching latent construct of sexual orientation, they do not always overlap.

Ex situ measures of sexual responding have often branched from self-reports in sexological research. For example, research on genital responding in women has concluded that heterosexual-identified women typically display a gender-nonspecific pattern (Chivers, 2017; though see Spape et al., 2014), and that bisexual-identified women typically respond more strongly to women (Bouchard et al., 2015; Chivers, 2017; Chivers et al., 2015; Timmers et al., 2015). While some have characterized branchedness between self-reports and genital responding as a product of their subjective and objective respective natures (Bailey et al., 2016), others have characterized this interpretation as biological reductionism, suggesting that branchedness could represent simple sexual diversity (van Anders, 2015). Regardless, these findings have proven to be extremely generative from a theoretical perspective, as demonstrated by Chivers (2017), who outlined ten hypotheses that aim to develop our empirical understanding of women’s sexual responses. Thus, ex situ measurement of sexual responding can be crucial to the scientific study of human sexuality.

As discussed, a range of ex situ measures of sexual orientation exist. While discussions in which these are compared often assume that they merely measure sexual orientation, with differences between measures being attributed to confounding, this is not necessarily so. Notably, models of sexual responding such as the information processing model of sexual arousal (Janssen et al., 2000) and the incentive motivation model of sexual motivation and behavior (Toates, 2009) conceptualize sexual responding as involving affective, cognitive, physiological, and behavioral processes, which trigger and influence one another. Integrating these two models, these processes consist of initial visual attention, implicit cognitive processing, later visual attention, explicit cognitive processing, affective processing, genital arousal, and subjective arousal, and different measures are thought to tap into each of these processes (Chivers, 2017). Just as it is not a given that these measures will always map onto one another, it is not a given that these processes will either. Indeed, as branchedness between self-report and genital responding could represent sexual diversity, this may be the case with branchedness between various ex situ measures, and identifying cases of branchedness could be theoretically generative. Thus, there is value in developing and applying a plurality of measures of ex situ sexual responding to a variety of research participants.

One specific ex situ measure that has shown some promise in men is the Implicit Relational Assessment Procedure (IRAP; Timmins et al., 2016). Timmins et al. demonstrated that a version of the IRAP that used nude images of women and men, paired with words relating to sexual attraction and aversion, was able to differentiate perfectly two small subsamples (*n* = 16 per group) of gay and heterosexual men from one another (area under the curve [AUC] = 1.00). Crucially, Timmins et al. also demonstrated that a high level of performance was maintained even when scores were calculated based solely on participants responses to images of men (AUC = 0.94) and women (AUC = 0.95). Based on this, Timmins et al. suggested that the IRAP may have utility as a measure of sexual orientation in bisexual men, and indeed perhaps in heterosexual, bisexual, and lesbian/gay women, though what aspect of sexual orientation was unclear. Additionally, a recent study explored heterosexual and lesbian women’s responses on a sexual orientation IRAP (da Silva et al., 2024). In this study, a different set of word stimuli and nude images were used to those used by Timmins et al. (2016), but the corresponding results were similar: Overall, male, and female *D*-IRAP scores significantly differentiated both groups from one another, albeit with lower precision (AUCs = 0.84-0.88). Thus, the IRAP has potential as a measure of sexual orientation in women as well.

As indicated by its name, the IRAP has historically been seen as an “implicit” measure, roughly similar to the Implicit Association Test (IAT; a task which also shown potential in the context of sexual orientation; Snowden & Gray, 2013; Snowden et al., 2008). Thus, the IRAP could be conceived as tapping into implicit cognitive processing, as outlined in the information processing model (Janssen et al., 2000). On balance, the IRAP was originally developed as a method for analyzing natural verbal relations from a relational frame theory perspective (Barnes-Holmes & Harte, 2022; Hussey et al., 2015; but see Hussey, 2022), and this approach has led to a Relational Frame Theory-based model of the behaviors typically produced on the IRAP (see Barnes-Holmes & Harte, 2022 for a recent review). From a sexological perspective, however, it may be useful, at least at this stage, simply to determine whether the IRAP may be used to predict aspects of sexual orientation and sexual responding, regardless of the more theoretical issues surrounding the procedure itself.

In line with previous literature on the IRAP (Barnes-Holmes et al., 2010a) and the information processing model (Janssen et al., 2000), Timmins et al. (2016) suggested that IRAP scores may predict relatively brief and/or automatic sexual responses, such as fleeting thoughts and fantasies and visual interest in certain gender/sex characteristics.^1^ On the other hand, self-report measures may tap into more volitional and/or extended sexual responses, such as strong sustained attractions and/or desires to have sex with a given person. Given that both the IAT and the IRAP require relatively rapid and brief responding, both may have some sensitivity to the more automatic processing of sexual stimuli. So, what distinguishes the IAT and the IRAP?

During an IAT, participants are asked to sort two sets of stimuli using one key (e.g., men and sex-associated words), and two related, but contrasting sets with another key (e.g., women and non-sex-associated words). At other times during the task, these pairings are flipped. Response latencies under these two conditions are measured and used to calculate a single overall score, which is thought to represent the relative strength of association between the pairings in memory (e.g., the relative strength of association with sex). In contrast, during the IRAP participants are presented with four trial types for each stimulus class pairing (e.g., men and synonyms for attractive; women and synonyms for attractive; men and synonyms for aversive; and women and synonyms for aversive). Participants are given response options (e.g., “true” and “false”). At different points in the task, participants are required to follow one of two opposing rules. For example, in Timmins et al. (2016) participants were asked to respond at some times as if all men were attractive and all women were unattractive, and at other times as if all women were attractive and all men were unattractive. Because the IRAP presents four individual trial types, researchers have historically calculated both relative and non-relative scores. Again, using Timmins et al. as an example, an overall score and separate scores for responses to the male and female stimuli were calculated and, as noted previously, the separate male and female IRAP scores differentiated heterosexual and gay men from one another with a high degree of precision.

At the time, Timmins et al. (2016) suggested that the IRAP had a key advantage over the IAT, in that it could output separate scores representing responding to men and women with high ability to differentiate gay and heterosexual men without resorting to multiple exposures to the different versions of the task. Research has since demonstrated that sexual orientation can indeed be measured non-relatively, but that participants indeed need to be exposed to three different iterations of the IAT (Snowden et al., 2020). Specifically, these three IATs used words associated with sex and not associated with sex, and they were differentiated by what pairs of stimulus sets they were sorted alongside: (1) images of nude men and women, (2) images of nude men and sexually neutral images, and (3) images of nude women and sexually neutral images. In this small sample study, heterosexual men (*n* = 32) responded quicker when sorting women and sex together than men and sex, gay men (*n* = 18) responded quicker when sorting men and sex together than women and sex, and the difference for bisexual men (*n* = 20) was non-significant. When exposed to the IATs which included neutral images, only the bisexual men responded significantly quicker both when sorting women and sex together and when sorting men and sex together than when sorting neutral images and sex together. Based on these preliminary results, the IAT appears to be a useful measure of men’s relative and absolute sexual associations with men and women, and these associations differ across heterosexual, bisexual, and gay men, though with the requirement of repeated exposure to the IAT to obtain all three measurements.

Slightly more complex results have been found for women. Snowden et al. (2024) recently exposed lesbian/gay (*n* = 43), bisexual (*n* = 48), and heterosexual (*n* = 78) women to these three versions of the IAT. They found that both lesbian/gay and bisexual women responded faster when sorting women and sex together than when sorting men and sex together, but that the difference for heterosexual women was non-significant. Notably, a lack of category-specific responding to nude images of men and women is common in ex situ measures of sexual responding (Chivers et al., 2010) and other measures of sexual responding also commonly find that bisexual women respond more strongly to female stimuli than to male (Bouchard et al., 2015; Chivers, 2017; Chivers et al., 2015; Timmers et al., 2015). However, all three groups responded significantly quicker both when sorting women and sex together and when sorting men and sex together than when sorting neutral images and sex together, which suggests that all three groups of women process both men and women as sexually attractive, or perhaps that the measure may instead be tapping into an association with sex more broadly defined in this group. Regardless, the IAT appears to be a useful measure of women’s relative sexual associations with men and women.

In summary, both the IRAP and the IAT represent promising potential measures of sexual orientation. The IRAP may have a key advantage over the IAT of providing non-relative scores with only one exposure. On balance, the IAT has shown promise in heterosexual, bisexual, and lesbian/gay men and women, whereas the IRAP has only been tested in one sample of heterosexual and gay men and one sample of heterosexual and lesbian/gay women. If the IRAP is to be used more widely in sexological research, it will be important to replicate the results reported by Timmins et al. (2016) and also to determine how women of various sexual orientations and bisexual men perform on the measure. Thus, the aim of the present study was to replicate the results of Timmins et al. (2016) and to determine how women of various sexual orientations and bisexual men perform on the measure. Additionally, while da Silva et al.’s (2024) results were not available when the present study was developed, our study also serves as a conceptual replication of the IRAP portion of that study. In the present study, an IRAP identical to that employed by Timmins et al. was presented to groups of heterosexual, lesbian/gay, and bisexual women and men.

We made the following predictions. Based on the above literature, we made these predictions separately for both men and women.

### Hypothesis 1

Overall *D*-IRAP Score will be associated with self-reported relative sexual attraction to men and women.

### Hypothesis 2

Male *D*-IRAP Score will be associated with self-reported sexual attraction to men.

### Hypothesis 3

Female *D*-IRAP Score will be associated with self-reported sexual attraction to women.

### Hypothesis 4

Overall *D*-IRAP Score will be associated with self-reported relative sexual contact with men and women.

### Hypothesis 5

Male *D*-IRAP Score will be associated with self-reported sexual contact with men.

### Hypothesis 6

Female *D*-IRAP Score will be associated with self-reported sexual contact with women.

### Hypothesis 7

Overall, Female and Male *D*-IRAP Scores will significantly differentiate gynesexual^2^ and androsexual

### Hypothesis 8

Overall and Male *D*-IRAP Scores will significantly differentiate heterosexual and bisexual participants.

### Hypothesis 9

Overall and Female *D*-IRAP Scores will significantly differentiate androsexual and bisexual.

## Method

### Participants

Participants were recruited using a wide range of on- and offline means, including in posts on Reddit, Facebook, and Twitter, advertisements on online dating sites/apps, and leaflets at sexual minority festivals and conventions. Both sexual minority-specific and general sites and apps were targeted. Potential participants read a detailed information sheet and, if they were interested in participating in the study, completed an online screening survey. The screening survey did not record data but allowed participants who reported meeting the following inclusion criteria to leave their contact information: (1) aged 18 or older, (2) fluent in English, (3) “normal” or “corrected to normal” vision, (4) identifies as a woman or a man, (5) identifies as heterosexual/straight, bisexual or gay/lesbian, and (6) minimum sexual and romantic history criteria contingent on sexual identity. Gynesexual participants were required to have had at least (6a) two female sexual partners, and (6b) one romantic relationship that lasted three months or more with a woman, androsexual participants were required to have had at least (6a) two male sexual partners, and (6b) one romantic relationship that lasted three months or more with a man, and bisexual participants were required to have had both. These criteria have previously been used to successfully screen out bisexual participants who did not have concordant responses on an ex situ measure of sexual responding (Rieger et al., 2005; Rosenthal et al., 2011). Participants were not excluded from participation based on the presence of previous romantic or sexual relationships, just the absence. Participants were not provided with an operational definition of sexual or romantic partner, and this was left to their own judgment.

We recruited 281 participants to the study. Despite originally reporting that they met the inclusion criteria in the screening survey, several participants reported information which conflicted with this. Such participants may originally have been rejected, then accessed the screening survey and gave new information, but then reverted to their original information in the study, so these participants were excluded. As such, we excluded 1 because they indicated that they were not fluent in English, 2 because they indicated that they did not have “normal” or “corrected to normal” vision, 16 participants because they did not meet the sexual and romantic history inclusion criteria as outlined below, and 1 participant because they did not answer the exclusion criteria questions (see Measures below).

One participant indicated that they identified as both a woman and genderqueer, so they were categorized as a woman for the purposes of the study. Four participants selected “Other sexual orientation” as their sexual identity. In the corresponding open field for this option (see Materials below), one participant specified that they use the term “homosexual,” so they were recoded as “gay/lesbian.” The other three participants either gave no elaboration or ambiguous elaboration, so were excluded from analyses. The remaining sample consisted of 44 lesbian/gay women, 42 bisexual women, 46 heterosexual women, 49 heterosexual men, 31 bisexual men, and 46 gay men, making for an initial total of 258 participants. A total of 24.4% of participants did not pass the performance criteria on at least two pairs of IRAP test blocks, and so our final numbers were 33 lesbian/gay women, 32 bisexual women, 34 heterosexual women, 35 heterosexual men,25 bisexual men, and 36 gay men, making for a total of 195 (99 women and 96 men). In terms of ethnicity/race, 144 participants were white, 23 participants were Asian, 20 participants were multiracial, 5 participants were Black and 3 participants were another ethnicity/race not listed. In terms of nativity, 134 participants were born in the UK, 60 were born in another country, and 1 had missing data. In terms of religion, 115 had no religion, 37 were Christian, and 43 had another religion. In terms of gender modality, 191 were cisgender and 4 were transgender.

### Procedure

Participation took place at a university in the UK. Upon arrival, participants were provided with a physical copy of the information sheet, identical to that with which they were provided at the time of the online screening. This sheet explained that the aim of the study was to investigate cognitive processes that are used in decisions that involve memory, specifically related to the sexual appeal of men and women. Participants were encouraged to ask questions and if they were still interested in taking part, they signed a consent form. Participants then completed two procedures, the order of which was randomized: (1) the survey and (2) the IRAP. Participants received £10.00 GBP for participation.

### Measures

#### Survey

Participants completed the survey on a laptop computer in a private room. An investigator waited outside while the participant completed the survey task.

Demographics: Participants were asked to report their age in years, the country or territory in which they were born, their religious affiliation, and their ethnic group.

Gender/Sex: Participants were asked how they identified their gender and provided with response options “Man (including transgender man, “FtM”),” “Woman (including transgender woman, “MtF”),” and “Other gender identity” with an area to specify what they meant if they selected the latter option. Participants were also asked what sex they were assigned at birth, meaning on their original birth certificate, and given options of “male” and “female.”

Sexual Identity: Participants were asked how they identify their sexual orientation and provided with response options: “Heterosexual (straight),” “Bisexual,” “Gay/Lesbian,” “Asexual,” and “Other sexual orientation” with an area to specify what they meant if they selected the latter option.

Sexual and Romantic History Screening: Across four questions, participants were asked to indicate the numbers of sexual partners that were men and women they had had during their entire lifetime, as well as the numbers of men and women with which they had had a romantic relationship that lasted three months or more. Response options were “0 (none)”, “1”, “2”, “3–5”, “6–10”, “11–20”, “21–50”, and “Over 50.” These items were based on those used in previous work that found that screening with these items helped select for bisexual men who displayed substantial sexual arousal to both men and women (Rosenthal et al., 2011).

Other Screening: Participants were asked if they were fluent in English and if they had “normal” or corrected to “normal” vision. Response options of “yes” and “no” were provided for each question.

Sell Assessment of Sexual Orientation: Participants completed the Sell Assessment of Sexual Orientation (Sell, 1996, 2011). The Sell Assessment consists of six pairs of matching questions, three of which ask about sexual attractions to men and women (e.g., “During the past year, how many different men were you sexually attracted to”), two ask about sexual behavior (e.g., “During the past year, how many different women did you have sexual contact with”), and one asks about sexual identity (“I consider myself”). Response options vary by question.

Kinsey (relative) summary scores were calculated for each pair of items as per the scale’s prescribed scoring (Sell, 1996, 2011). Overall sexual attraction scores were calculated by averaging the three attraction Kinsey summary scores (*α* = .99 in men, α = .96 in women) and overall sexual contact scores were calculated by averaging the two contact Kinsey summary scores (*r*_Spearman-Brown_ = .99 in men, *r*_Spearman-Brown_ = .99 in women). Kinsey summary scores of 0 (asexual), e.g., for participants who had no sexual contact in the past year, were treated as missing data for this purpose. Men’s overall sexual attraction and sexual contact scores were reversed so that lower scores represented higher, or more exclusive, androsexuality and higher scores represented higher, or more exclusive, gynesexuality for both men and women, thus reflecting overall *D*-IRAP scores (see below). Thus, regardless of participant gender, a score of 1 represented exclusive sexual attraction to (or sexual contact with) men during the past year, a score of 7 represented exclusive sexual attraction to (or sexual contact with) women during the past year, and a score of 4 represented approximately equal attraction to (or contact with) men and women over the past year.

Female sexual attraction scores were calculated by totaling the three corresponding items and transforming to a 1–7 scale (*α* = .94 in men, *α* = .90 in women). Female sexual contact scores similarly were calculated by totaling the two corresponding items and transforming to a 1–7 scale (*r*_Spearman-Brown_ = .72 in men, *r*_Spearman-Brown_ = .69 in women). Higher scores represented higher gynesexuality, reflecting female *D*-IRAP scores. Regardless of participant gender, a female attraction (or contact) score of 1 represented no attraction to (or no contact with) women during the past year, whereas a score of 7 represented maximum attraction to (or contact with) women during the past year.

Male sexual attraction scores (*α* = 0.92 in men, *α* = 0.97 in women) and male sexual contact scores (*r*_Spearman-Brown_ = 0.85 in men, *r*_Spearman-Brown_ = 0.69 in women) were calculated in the same way as female sexual attraction and sexual contact scores, respectively, with the addendum that male scores were reversed such that lower scores represented higher androsexuality, reflecting male *D*-IRAP scores. Thus, regardless of participant gender, a male attraction (or contact) score of 1 represented maximum attraction to (or contact with) men during the past year and a score of 7 represented no attraction to (or no contact with) men during the past year.

Other Measures: Measures of sexual arousal, sexual disgust, sexual curiosity, relationship status, employment status, and yearly income in GBP were also included in the survey. These were not used in the present study but were taken for potential use in future research. These have been mentioned here for completeness.

#### Implicit Relational Assessment Procedure

Participants completed the IRAP on the same laptop computer and in the same private room as the survey. An investigator waited outside, while the participant completed the IRAP. The IRAP software began by presenting a set of instructions, which explained the IRAP task using illustrative examples of the different types of trials and giving a detailed account of what participants were required to do. Participants were instructed to come out and ask the investigator if they had any questions.

**Fig. 1:**
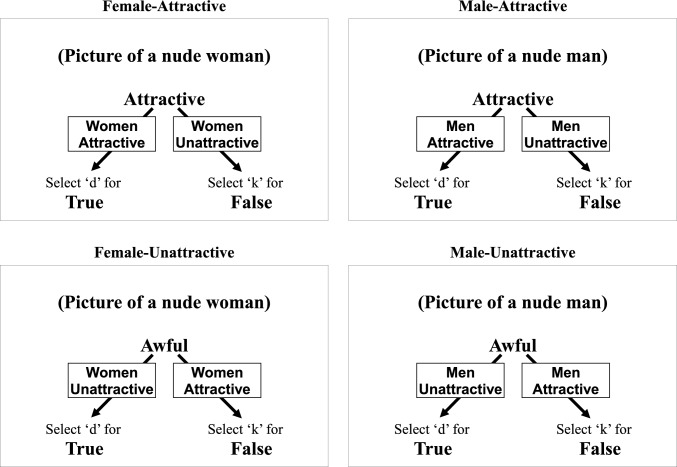
Examples of the four IRAP trial types. The nude picture stimuli, word stimuli, and response options (“True” and “False”) appeared simultaneously on each trial. Arrows with superimposed text show which responses indicate which bias (text and arrows did not appear on screen)

The IRAP was presented in blocks of 40 trials. Trials consisted of the simultaneous presentation of either a male or female nude picture stimulus at the top of the screen, either an attractive or unattractive word stimulus in the middle of the screen and response options of “True’’ and ‘‘False’’ in the bottom left- and right-hand corners, with the instructions ‘‘Press ‘D’ for’’ and ‘‘Press ‘K’ for’’ directly above the left and right response options, respectively. The left–right positioning of the two response options, and therefore the keys required to select them varied randomly across trials, with the constraint that they could not appear in the same positions across more than three successive trials. The different combinations of male/female and positive/negative words resulted in four possible trial types: Male-Attractive, Male-Unattractive, Female-Attractive, and Female-Unattractive (see Fig. 1).

During each block, participants had to respond in accordance with one of two rules, regardless of their own personal feelings: (1)‘‘all females are attractive and all males are unattractive’’(defined as a female-attractive block) or (2) ‘‘all males are attractive and all females are unattractive’’ (defined as a male-attractive block). The trials were presented quasi-randomly with the constraint that each of the four trial types appeared 10 times within each 40-trial block, all 10 picture and 10 word stimuli were presented twice within each block and the same trial type was not presented across successive trials.

Choosing the response option deemed correct cleared the screen for a 400-ms inter-trial interval and then the next trial was presented. If the incorrect response option was chosen, a red X appeared directly underneath the target word and remained there until the participant chose the correct response option. If a participant failed to respond within 2000 ms from the start of a trial the words ‘‘Too Slow’’ appeared toward the center bottom of the screen and remained there until the participant chose one of the response options.

Participants were first presented with a set of two practice blocks. Participants were required to achieve an accuracy criterion of ≥ 80% correct responses and a median response latency of ≤ 2000 ms. If these criteria were achieved, participants were then exposed to a fixed set of six test blocks. If they were not achieved, the practice blocks were repeated until they were. Participants were given the option of ending participation if they had trouble meeting the criteria during the practice blocks. Participants were not required to achieve any performance criteria during the test blocks in order to proceed. However, accuracy and latency feedback were presented at the end of each block to encourage participants to maintain the performance criteria achieved during the practice blocks.

Consistent with the majority of published IRAP studies, individual response latency data were transformed into *D*-IRAP scores (see Barnes-Holmes, Barnes-Holmes, et al., 2010a) using an adaptation of the Greenwald et al. (2003) *D*-algorithm. The full process is outlined in Appendix A. Note that data were retained for participants who maintained performance criteria across at least two pairs of test blocks. In the case of participants who did not maintain performance criteria in one pair of test blocks, scores were calculated based on the other two pairs of test blocks only. This diverges with most previous work, but maximizes data retention and has been used previously, specifically with the Sexual Orientation IRAP (Timmins et al., 2016).

The *D*-algorithm produces a *D*-IRAP score for each of the four trial types. For the two female trial type scores, a positive score indicates an attraction bias, and a negative score indicates an aversion bias, whereas for the two male trial type scores a negative score indicates an attraction bias, and a positive score indicates an aversion bias. The mean of the two female trial type scores constitutes the female pictures *D*-IRAP score, and the mean of the two male trial type scores constitutes the male pictures *D*-IRAP score. The mean of the four trial types scores constitutes the overall mean *D*-IRAP score. A positive score thus indicates a gynesexual bias (i.e., stronger attraction to female than male pictures), whereas a negative score indicates an androsexual bias (i.e., stronger attraction to male than female pictures).

#### Stimuli

The same ten male and female picture stimuli used by Timmins et al. (2016) taken from the International Affective Picture System (IAPS; Bradley & Lang, 2007) were employed in the current study (male picture numbers: 4460, 4500, 4534, 4550, 4561; female picture numbers: 4141, 4142, 4210, 4235, 4240). All pictures chosen by Timmins et al. (2016) were picked for their erotic, but not pornographic content; subjects in the pictures were completely or almost completely nude, while not visibly sexually aroused nor engaged in sexual activity. Nine of these pictures were also used by Snowden et al. (2008) in a similar IAT. Three of the female stimuli and two of the male stimuli had parts of their body covered by clothing or a towel. The breasts and mons pubis of all female stimuli were visible, though the genitalia themselves were obscured by angle of view or pubic hair for all female stimuli. The upper torso was visible for all male stimuli and the genitalia were visible for three of the male stimuli. To maintain novelty and efficacy of the IAPS images, they are not allowed to be published, but readers can obtain access based on the instructions in Bradley and Lang (2007).

The ten word stimuli pertaining to “sexually attractive” (i.e., “arousing,” “erotic,” “attractive,” “sensual,” and “exciting”) and “sexually unattractive” (i.e., “repulsive,” “repelling,” “repugnant,” “repellent,” and “awful”) used by Timmins et al. (2016) were also employed in the current study. Nine of these words were also used by Snowden et al. (2008) in a similar IAT.

### Data Analysis

For all hypotheses, we set an alpha level of 0.05. To address Hypotheses 1–6, we calculated Pearson’s correlation coefficients with 95% confidence intervals for both men and women. To address Hypotheses 7–9, we used the same signal detection test employed by Timmins et al. (2016), which involved constructing the Receiver Operator Characteristic (ROC). This method was chosen as ROC analysis provides a greater level of detail than other difference tests through its comprehensive visualization for discriminating between cases, which is particularly suited to categorical testing (Nahm, 2022) and to allow for comparability with the results of Timmins et al. (2016).

A ROC is a graph in which the probability of a true positive, or a “hit,” is plotted against the probability of a false positive or a “false alarm” (Fawcett, 2006). From this, the AUC can be calculated, which is the statistical likelihood that a randomly chosen member of one group will have a higher score than a randomly chosen member of another group. Therefore, a test with perfect ability to predict group membership would have an AUC = 1.0, and a test with no ability to detect group membership would have an AUC = 0.5. Thus, the null value for null hypothesis significance testing is AUC = 0.5. 95% confidence intervals for the AUC were also calculated.

## Results

**Fig. 2:**
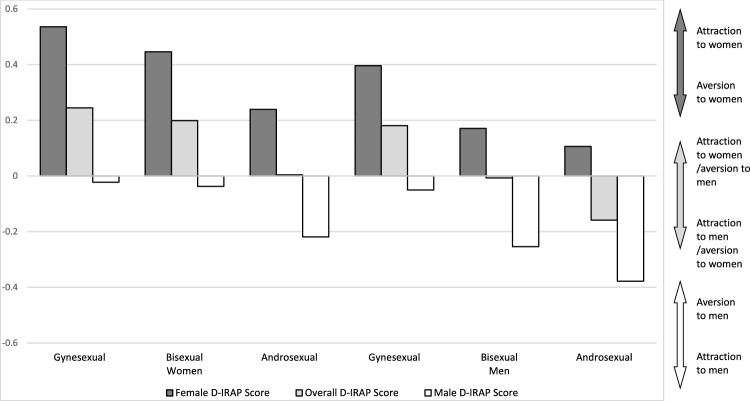
Median *D*-IRAP scores for female picture and male picture trial types for gynesexual, bisexual, and androsexual participants. For female *D*-IRAP scores, higher scores indicate a positive bias (attraction) and lower scores indicate a negative bias (aversion). For male *D*-IRAP scores, lower scores indicate a positive bias (attraction) and higher scores indicate a negative bias (aversion). For overall *D*-IRAP scores, higher scores indicate a positive bias (attraction) toward women and/or a negative bias (aversion) toward men, whereas lower scores indicate a positive bias (attraction) toward men and/or a negative bias (aversion) toward women

Data were screened for univariate outliers prior to analyses. No outliers were detected for male participants. A univariate outlier was detected by calculating z scores for each variable and using cutoffs of ± 3.29 (Tabachnick & Fidell, 2013) for the Female *D*-IRAP variable, *z* = 3.61, and for a separate case for the Male *D*-IRAP variable, *z* = − 3.30. Both scores belonged to female participants. These entries were manually checked, and it was determined they were valid responses unaffected by entry or calculation errors. *D*-IRAP scores broken down by gender and sexual orientation are presented in Table 1 and Fig. 2.

**Table 1:** Descriptive statistics for gynesexual, bisexual, and androsexual women and men’s ages, *D*-IRAP scores, and self-reported sexual attraction and sexual contact scores

	Women			Men		
	Gynesexual	Bisexual	Androsexual	Gynesexual	Bisexual	Androsexual
*n*	33	32	34	35	25	36
	Mdn (IQR)	Mdn (IQR)	Mdn (IQR)	Mdn (IQR)	Mdn (IQR)	Mdn (IQR)
*D*− IRAP Score						
Overall	.24 (.12, .46)	.20 (.05, .39)	.00 (− .16, .15)	.18 (.08, .33)	− .01 (− .11, .18)	− .16 (− .34, .05)
Female	.54 (.38, .74)	.45 (.24, .65)	.24 (.05, .41)	.40 (.21, .71)	.17 (.03, .47)	.11 (− .15, .28)
Male	− .02 (− .18, .24)	− .04 (− .25, .19)	− .22 (− .44, − .03)	− .05 (− .16, .14)	− .25 (− .33, − .04)	− .38 (− .64, − .15)
	*M (SD)*	*M (SD)*	*M (SD)*	*M (SD)*	*M (SD)*	*M (SD)*
*D*− IRAP Score						
Overall	.29 (.23)	.19 (.26)	− .02 (.19)	.23 (.21)	.01 (.18)	− .16 (.21)
Female	.55 (.24)	.42 (.31)	.22 (.23)	.45 (.26)	.22 (.27)	.07 (.26)
Male	.03 (.32)	− .04 (.29)	− .24 (.26)	.01 (.26)	− .21 (.18)	− .40 (.28)
Sexual Attraction						
Overall^a^	6.46 (.78)	4.15 (1.07)	2.01 (1.01)	6.73 (.48)	3.72 (.76)	1.39 (.61)
Female^b^	4.88 (1.00)	4.89 (1.17)	2.05 (1.06)	5.53 (1.15)	5.46 (1.17)	1.52 (.86)
Male^c^	6.40 (1.05)	3.31 (1.47)	3.21 (.80)	6.68 (.59)	2.12 (.84)	2.04 (1.05)
Sexual Contact						
Overall^a^	6.63 (.94)	3.52 (2.31)	1.14 (.58)	7.00 (.00)	2.92 (1.55)	1.04 (.26)
Female^b^	2.80 (.92)	2.30 (1.00)	1.05 (.22)	3.04 (.84)	2.33 (1.23)	1.03 (.15)
Male^c^	6.80 (.54)	5.14 (1.35)	4.81 (.78)	7.00 (.00)	4.84 (.81)	4.14 (1.17)
Age	34.5 (11.7)	29.1 (10.1)	26.4 (5.4)	27.7 (8.1)	37.1 (13.9)	32.5 (12.3)

For all measures, overall scores were calculated using responses toward both women and men; higher Overall scores indicate higher, or more exclusive, gynesexuality, and lower overall scores indicate higher, or more exclusive, androsexuality. Female scores were calculated using only responses toward women; higher Female scores indicate higher gynesexuality. Male scores were calculated using only responses toward men; lower Male scores indicate higher androsexuality

^a^1 = exclusive sexual attraction to (or sexual contact with) men during the past year, 4 = approximately equal sexual attraction to (or sexual contact with) men and women over the past year, 7 = exclusive sexual attraction to (or sexual contact with) women during the past year

^b^1 = no sexual attraction to (or no sexual contact with) women during the past year, 7 = maximum sexual attraction to (or sexual contact with) women during the past year

^c^1 = maximum sexual attraction to (or sexual contact with) men during the past year, 7 = no sexual attraction to (or no sexual contact with) men during the past year

Pearson correlation coefficients and 95% confidence intervals are shown in Table 2. Missing data ranged from 1.0 to 6.25% on self-report scores. Pairwise deletion was used for the purposes of these analyses. Except for women’s attraction scores for men, all *D*-IRAP scores were significantly associated with their self-report counterparts, supporting Hypotheses 1–6 for men and Hypotheses 1 and 5–6, but not Hypothesis 2, for women. As a brief reminder, Hypothesis 1–3 predicted that the overall, male, and female *D*-IRAP Scores would be associated with their corresponding self-reported sexual attraction scores, and Hypothesis 2 was that Male *D*-IRAP Scores would be associated with self-reported sexual attraction to men. Hypotheses 4–6 predicted that the overall, male, and female *D*-IRAP Scores would be associated with their corresponding self-reported sexual behavior scores.

**Table 2:** Pearson’s *r* and 95% confidence intervals for IRAP scores and Sell assessment of sexual attraction and contact counterparts

Variable	Overall *D*-IRAP Score		Female *D*-IRAP Score		Male *D*-IRAP Score	
	*r* [95% CI]	*p*	*r* [95% CI]	*p*	*r* [95% CI]	*p*
Women						
Kinsey Attraction—Overall	**.50 [.34, .64]**	**< .001**				
Kinsey Contact—Overall	**.53 [.36, .66]**	**< .001**				
Attraction—Female			**.48 [.31, .62]**	**< .001**		
Contact—Female			**.45 [.27, .59]**	**< .001**		
Attraction—Male					.16 [− .04, .35]	.115
Contact—Male					**.24 [.05, .42]**	**.016**
Men						
Kinsey Attraction—Overall	**.64 [.51, .75]**	**< .001**				
Kinsey Contact—Overall	**.63 [.48, .74]**	**< .001**				
Attraction—Female			**.48 [.30, .62]**	**< .001**		
Contact—Female			**.38 [.19, .53]**	**< .001**		
Attraction—Male					**.45 [.28, .60]**	**< .001**
Contact—Male					**.50 [.33, .64]**	**< .001**

Bolded = Significant

Pearson correlation is sensitive to outliers (de Winter et al., 2016). As such, we performed sensitivity analyses for all correlations which involved the female and male *D*-IRAP scores in women by running these again with the respective outlier cases excluded. Female *D*-IRAP Score remained significantly associated with both self-reported sexual attraction, *r* = .50, 95% CI[.33, .64], *p* < .001 and self-reported sexual contact, *r* = .44, 95% CI[.27, .59], *p* < .001. Male *D*-IRAP Score remained significantly associated with self-reported sexual contact, *r* = .22, 95% CI[.02, .40], *p* = .031 and non-significantly associated with self-reported sexual attraction, *r* = .12, 95% CI[− .08, .31], *p* = .245. These were similar values, suggesting that these findings were robust to the inclusion of the outliers.

**Fig. 3:**
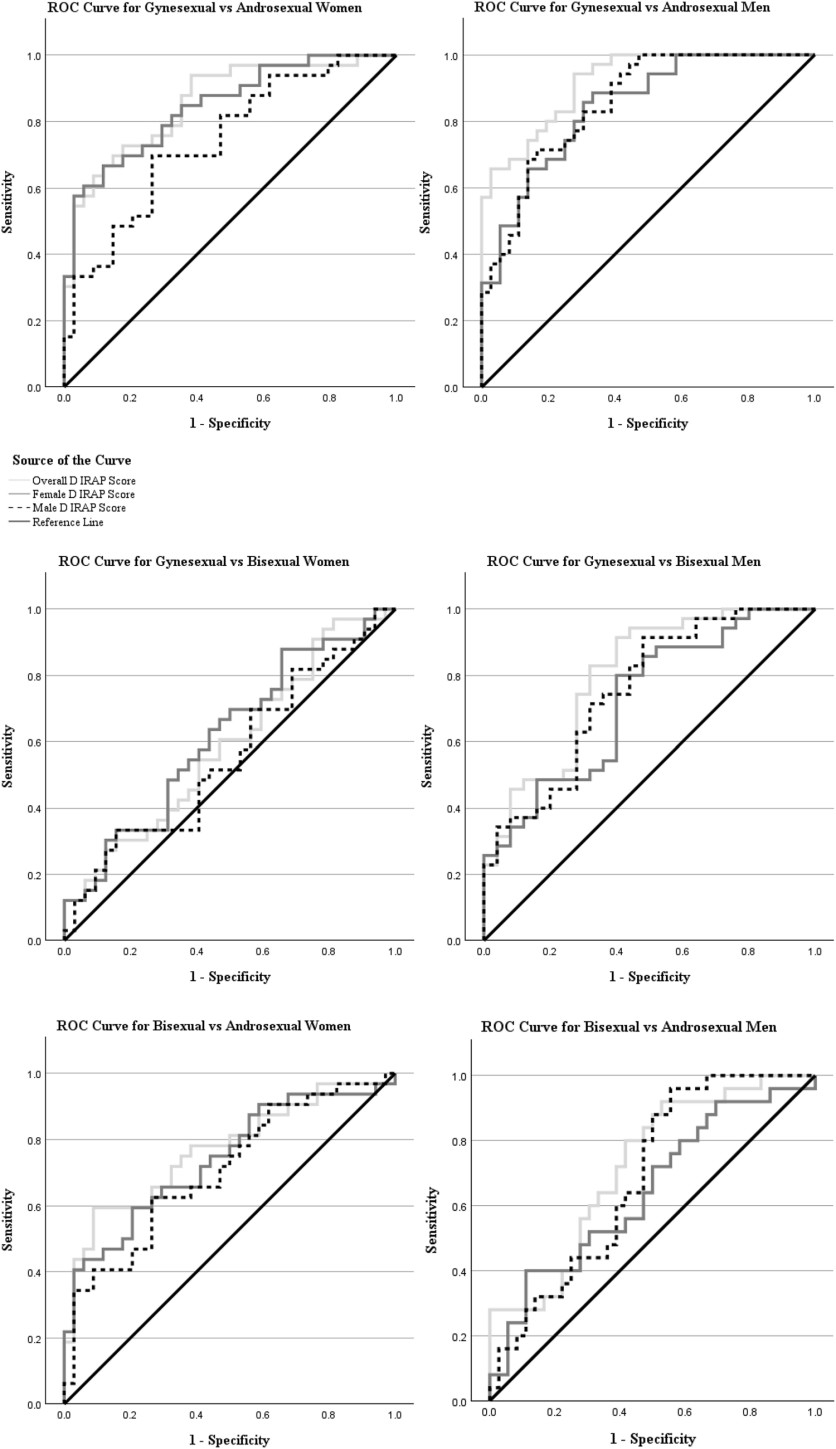
Receiver operating characteristics of the ability of the overall, female and male *D*-IRAP scores to predict sexual orientation between gynesexual, bisexual, and androsexual women and men

ROC curves are presented in Fig. 3, and AUCs and 95% confidence intervals are shown in Table 3. These were initially performed based on self-reported identity. All *D*-IRAP scores had a significant predicative ability for each sexual orientation pairing except for heterosexual and bisexual women, supporting Hypotheses 7–9 for men and Hypotheses 7 and 9, but not 8, for women. As a brief reminder, Hypothesis 7 was that overall, female and male *D*-IRAP Scores would significantly differentiate gynesexual and androsexual participants, Hypothesis 8 was that overall and male *D*-IRAP scores would significantly differentiate gynesexual and bisexual participants, and Hypothesis 9 was that overall and female *D*-IRAP scores would significantly differentiate androsexual and bisexual participants. ROC analysis and AUC are not affected by outliers and so we did not perform any sensitivity analyses.

**Table 3:** Areas under the curve and 95% confidence intervals for IRAP scores comparing gynesexual, androsexual and bisexual women and men

Variable	Overall *D*-IRAP Score		Female *D*-IRAP Score		Male *D*-IRAP Score	
	*AUC* [95% CI]	*p*	*AUC* [95% CI]	*p*	*AUC* [95% CI]	*p*
Women						
Gynesexual vs Androsexual	**.86 [.77, .95]**	**< .001**	**.85 [.76, .94]**	**< .001**	**.74 [.63, .86]**	**< .001**
Gynesexual vs Bisexual	.59 [.45, .73]	.213	.61 [.48, .75]	.101	.56 [.41, .69]	.445
Bisexual vs Androsexual	**.77 [.65, .88]**	**< .001**	**.74 [.62, .86]**	**< .001**	**.71 [.58, .83]**	**.001**
Men						
Gynesexual vs Androsexual	**.92 [.86, .98]**	**< .001**	**.84 [.76, .93]**	**< .001**	**.85 [.77, .94]**	**< .001**
Gynesexual vs Bisexual	**.80 [.68, .91]**	**< .001**	**.72 [.59, .85]**	**.001**	**.75 [.63, .88]**	**< .001**
Bisexual vs Androsexual	**.72 [.59, .85]**	**.001**	**.64 [.50, .79]**	**.046**	**.69 [.55, .82]**	**.006**

AUC = Area Under the Curve

Bolded = Significant (*α* = 0.05)

After the analyses were complete, a reviewer suggested that as sexual attractions do not always map onto sexual identities, identity may not be an ideal means of categorizing our participants for the purposes of our ROC analyses. While there are strong reasons to use identities in these types of analyses, based on this feedback we created lesbian/gay, bisexual and heterosexual categories using the overall sexual attraction scores from the Sell Assessment in order to perform sensitivity analyses. Participants who scored 1–2.5 were classed as androsexual, participants who scored 2.5–5.5 were classed as bisexual, and participants who scored 5.5–7 were classed as gynesexual. These roughly map onto Kinsey 0–1 for heterosexual, Kinsey 2–4 for bisexual, and Kinsey 5–6, in line with many previous studies (e.g., Rieger et al., 2005). Corresponding categories and analyses relating to sexual contacts were not created as participants were already stratified by sexual contacts based on the inclusion criteria.

With regard to men, based on these scores, 33 heterosexual-identified men remained classified as heterosexual, 25 bisexual-identified men remained classified as bisexual, and 33 gay men remained classified as gay. A total of 2 heterosexual-identified men and 2 gay-identified men were reclassified as bisexual. No bisexual-identified men were reclassified, and 1 man was dropped for the purpose of these analyses due to missing self-report data. This made for sample sizes of 33 heterosexual men, 29 bisexual men, and 33 gay men (total 95 men). This recategorization did not change the pattern of results of our ROC analyses, suggesting that these results are robust to the selected means of sexual orientation categorization. These results are presented in Appendix B.

With regard to women, based on these scores, 22 heterosexual-identified women remained classified as heterosexual, 27 bisexual-identified women remained classified as bisexual, and 29 lesbian/gay-identified women remained classified as lesbian/gay. A total of heterosexual-identified women and 3 lesbian/gay-identified women were reclassified as bisexual, 3 bisexual-identified women were reclassified as lesbian, and 1 bisexual-identified woman was reclassified as heterosexual. Just 3 women were dropped for the purpose of these analyses due to missing self-report data. This made for samples sizes of 23 heterosexual women, 41 bisexual women, and 32 lesbian/gay women (total 96 women). The majority of results remained the same in terms of significance; however, lesbian/gay and bisexual women became significantly differentiated by the overall (AUC = .70, 95% CI[.58, .82], *p* = .001) and female (AUC = .73, 95% CI[.61, .84], *p* < .001) *D*-IRAP scores, and bisexual and heterosexual women were no longer significantly differentiated by male *D*-IRAP scores (AUC = .60, 95% CI[.46, .75], *p* = .170). This suggests most ROC analyses for women were robust to the selected means of sexual orientation categorization, but these three were not. These results are presented in Appendix B.

## Discussion

The present study investigated the relationships between a sexual orientation IRAP and self-report measures of sexual attraction and sexual behavior in heterosexual, bisexual, and gay men and women. In addition, the study tested how heterosexual, bisexual, and gay men and women differed on this sexual orientation IRAP. Significant correlations were found between all IRAP scores and all corresponding self-report measures for men, whereas for women there was a single exception: the *D*-IRAP score calculated using responses to images of men with self-reported sexual attraction to men. Similarly, all *D*-IRAP scores significantly predicted sexual orientation between all sexual orientations for men, whereas for women, all *D*-IRAP scores significantly predicted sexual orientation between lesbian/gay and heterosexual women, and between bisexual and heterosexual women, but not between bisexual and lesbian/gay women.

The results of the current study for men are in line with those of Timmins et al. (2016). All three *D*-IRAP scores had a strong ability to differentiate heterosexual and gay men from one another. These effects were slightly weaker than those found by Timmins et al. For example, in Timmins et al., the point estimates for AUCs suggested the overall *D*-IRAP score had a perfect ability to differentiate these two groups from one another, and the corresponding male and female scores had a near perfect ability. Similarly, the correlations found by Timmins et al. between men’s self-report measures and their *D-*IRAP scores were stronger, though this is not a perfect comparison for two reasons: (1) the sample used to calculate these in the present study included bisexual men, whereas Timmins et al.’s sample only included heterosexual and gay men, and (2) both studies used different self-report measures. Furthermore, all three *D*-IRAP scores also demonstrated an ability to differentiate bisexual men from both heterosexual and gay men. These findings collectively imply that the IRAP may be a good measure of sexual orientation in men, at least in terms of relative responses to men and women.

Significant correlations were also found in women between overall *D*-IRAP scores and corresponding self-report measures, between female *D*-IRAP scores and corresponding self-report measures, between Male *D*-IRAP scores and self-reported sexual contact with men, but not with self-reported sexual attraction toward men. Additionally, all three *D*-IRAP scores significantly differentiated heterosexual women from both bisexual women and lesbian/gay women. These results for heterosexual and lesbian/gay women are in line with the only other study in which women completed a sexual orientation IRAP (da Silva et al., 2024), though notably in that study all *D*-IRAP scores significantly correlated with the Klein Sexual Orientation Grid, a multidimensional self-report measure of sexual orientation. As with the present study’s correlations in men, comparison of the present study’s correlations in women are imperfect due to the inclusion of bisexual participants and the use of a different measure.

In the present study, there was no evidence that any of the three *D*-IRAP scores could differentiate bisexual women from lesbian/gay women, at least when using self-reported identity. It would be a misunderstanding of null hypothesis significance testing to argue that this is evidence that the *D*-IRAP cannot differentiate bisexual and lesbian/gay women or that it is better at differentiating bisexual and lesbian/gay women than bisexual and heterosexual women (Gelman & Stern, 2006; Greenland, 2012). Indeed, the sensitivity analyses suggested that this finding may just be an artifact of our use of identity to categorize participants rather than categorizing them based on their self-reported sexual attraction to men and women. Nevertheless, this finding contrasts with our expectations and is worthy of further inquiry; future research using a larger sample capable of equivalence testing (Lakens et al., 2018) should examine whether *D*-IRAP scores are unable to differentiate bisexual and lesbian/gay women. If the IRAP is indeed incapable of this differentiation, it may be that in women, the IRAP only taps into sexual responses to other women and does not tap into sexual responses to men, at least when using this set of stimuli.

Notably, the finding that the male *D*-IRAP scores significantly predicted sexual orientation for bisexual vs heterosexual women was not robust to how participants were categorized; when participants were categorized based on self-reported patterns of sexual attraction, male *D*-IRAP scores did not significantly predict sexual orientation between these groups. On the other hand, when participants were categorized in this manner, the overall and female *D-*IRAP scores significantly predicted sexual orientation for lesbian/gay vs bisexual women. Given the small sample size, it is impossible to say whether this represents a genuine difference in performance of the IRAP based on how female participant’s sexual orientation is coded, and arguments to that effect, once again, would be on the basis of a fundamental misunderstanding of null hypothesis significance testing (Gelman & Stern, 2006; Greenland, 2012). However, future research with larger sample sizes should investigate whether the sexual orientation IRAP taps into identity, sexual attraction, or both.

To further contextualize the results in women, it is worth revisiting research on the specificity of sexual responding. As mentioned, such research frequently concludes that heterosexual-identified women show a gender-nonspecific pattern of sexual responding (Chivers, 2017; though see Spape et al., 2014) and bisexual-identified women typically respond more strongly to female stimuli than to male stimuli (Bouchard et al., 2015; Chivers, 2017; Chivers et al., 2015; Timmers et al., 2015). Research on lesbian/gay-identified women has found they also respond more strongly to female stimuli than to male stimuli (Micanovic et al., 2021). This pattern has also been observed in the IAT (Snowden et al., 2024) and the IRAP (da Silva et al., 2024). In the present study, observed median overall *D*-IRAP scores for heterosexual women were at 0, implying this group responded about as fast during both block types, whereas observed overall *D*-IRAP scores for bisexual and lesbian/gay women were positive, implying that they responded faster during female-attractive blocks than during male-attractive. Originally, we did not hypothesize this pattern of results, as research has suggested that the midpoint for a *D*-IRAP score is not necessarily zero, due to the potential influence of spurious variables, such as those associated with the use of specific response options (Barnes-Holmes et al., 2010a, 2010b; Hussey & Drake, 2020). Practically, this means that between-group comparisons are typically useful, but within-group comparisons against a hypothetical null of zero are not. At the same time, the striking similarity between this pattern of results and what generally appears in the literature for women, as well as for men, suggests that future research should explore whether scores generated by the sexual orientation IRAP correspond with other ex situ measures of sexual responding, such as the IAT or traditional, physiological measures.

Future research should also investigate whether variables unrelated to sexual orientation are associated with sexual orientation *D*-IRAP scores. For example, women who have high body image concerns and are quite concerned with what constitutes an attractive woman could respond faster than women who are lower in such concerns when expected to respond as if all women in this study are attractive due to the stimuli representing women who are considered conventionally attractive. To some degree, this would reflect Chivers’ (2017) proposal that the ubiquitous sexual objectification of women’s bodies as outlined in objectification theory (Fredrickson & Roberts, 1997) may influence women’s responses on ex situ sexual orientation measures. However, in the case of the IRAP it is possible that the responses could reflect body image concerns even if the sexual response system is not activated. This, combined with the protective hypothesis (Dahlenburg et al., 2020), could also potentially explain the results for women in the present study. Specifically, the protective hypothesis posits that lesbians are less influenced by heteronormative beauty standards and unconcerned with attracting men and are thus somewhat protected from heteronormative cultural norms regarding women’s bodies. Bisexual and heterosexual, but not lesbian/gay, women’s *D*-IRAP scores may be shifted toward gynesexuality, resulting in heterosexual women’s responses to male and female stimuli being equal, bisexual women’s responses being biased toward female over male stimuli, and *D*-IRAP scores failing to differentiate lesbian/gay and bisexual women from each other. This can only be confirmed or refuted by further research.

Of note, male *D*-IRAP scores significantly differentiated heterosexual women from bisexual women and gay men from bisexual men, despite all these groups self-reporting a sexual orientation which includes men. This suggests that male *D*-IRAP scores are predictive of sexual orientation toward women, despite only being calculated using responses toward men. Similarly, female *D*-IRAP scores significantly differentiated heterosexual men from bisexual men, despite both groups self-reporting an orientation which includes women. Arguably, this contrasts with suggestions by Timmins et al.’s (2016) that the IRAP may be able to measure sexual responding to men and women separately. Despite the male and female *D*-IRAP scores being generated using responses to their respective trial types, these scores appear to have been influenced by participants’ self-reported attraction to the other gender. Put another way, the male and female *D*-IRAP scores have demonstrated poor discriminant validity. It is an open question whether sexual responses toward males and females are truly independent (Jiang et al., 2006), and thus, whether interdependence between the scores would be expected. One would at least expect, for example, that female *D*-IRAP scores would differentiate bisexual and androsexual participants more effectively than male *D*-IRAP scores. Despite this, point estimates for these comparisons were similar for male and female *D*-IRAP scores in both groups. Future work with larger samples will be required to determine whether these scores do measure sexual responding to men and women separately or whether they are roughly equivalent to the overall score.

The fact that male and female *D*-IRAP scores appear to be interdependent reflects a more recent conceptual and empirical analysis of the IRAP known as the Differential Arbitrarily Applicable Relational Responding Effects (DAARRE) model (Finn et al., 2018) and a recent review of the IRAP as a general measure, which found that scores calculated from different IRAP trial types are not entirely independent, though there is a variation across domains (Hussey, 2023a). In practical terms, this means that gender-specific *D*-IRAP scores derived from the sexual orientation IRAP should be interpreted with caution until a solution to the IRAP’s relativity issues may be identified and validated. For most of the sexual orientation, IRAP’s uses this is not a fatal issue. As with the sexual orientation IAT, the overall score can be used and interpreted. However, it does suggest that the sexual orientation IRAP may not readily differentiate specific groups of individuals for whom relative responses to men and women would be expected to be similar, for example bisexual and asexual individuals. In the event a solution is not found, this issue could potentially be resolved with multiple IRAPs in the same manner as the IAT (Snowden et al., 2020).

Posterior to the conceptualization of the present study, and so not forming a basis for its aims or hypotheses, a paper was published in which the DAARRE model was outlined in the context of sexual orientation (da Silva et al., 2024). Describing this module in detail would be beyond the scope of the current paper, but put simply, da Silva et al. suggest that sexual orientation IRAP scores may represent a combination of both sexual orientation and attitudinal learning history. This accords with the present study’s results in women and the interpretation that women’s responses were shifted toward female stimuli due to societal sexual objectification of women’s bodies (Chivers, 2017; Fredrickson & Roberts, 1997).

Notably, while some previous research using ex situ sexual orientation measure has been interpreted as providing evidence for or against the existence of certain sexual orientations, particularly bisexuality in men and heterosexuality in women, such interpretation of our findings is discouraged. As discussed, sexual responding is conceived as involving several different processes which different measures may tap into. It is not a given that responses to gender/sex stimuli on the IRAP will map onto other measures of sexual orientation, let alone that sexual orientation can be reduced to such responses. Indeed, interpretations of research findings that reduce sexual orientation to a single ex suite measure have been criticized as overstating implications (Feinstein & Galupo, 2020; Zivony, 2020). The present study was also unable to answer which specific processes the IRAP taps into. This corroborates the need for future research which explores whether scores generated by the sexual orientation IRAP correspond with other ex situ measures of sexual responding, such as the IAT or traditional, physiological measures. Such research could also help tease out what aspects of sexual orientation and sexual responding the IRAP’s scores tap into and indeed identify theoretically generative discrepancies between measures.

A key strength of this study is that we collected data across heterosexual, bisexual, and lesbian/gay men and women using the same implicit sexual orientation measure, stimuli, and explicit measures. To our knowledge, this is the first study in which this has been done. This increases the comparability of our results across participant groups by reducing the risk that extraneous variables such as stimuli or time-related confounds could be responsible for differences between groups.

This study, of course, also suffers from its limitations. Firstly, data were not collected on where participants were recruited, preventing us from investigating this as a potential explanation for any non-significant findings. Furthermore, there were two interlinked limitations, attrition and sample size. Starting with attrition, a quarter of participants did not reach the IRAP’s accuracy and/or performance criteria. This is common for IRAP research, likely owing to the complexity of the task (Cabrera et al., 2021), and indeed for research using ex situ measures of sexual orientation. For example in Rieger et al. (2005), 32.7% of participants’ phallometric data were discarded due to having insufficient genital arousal. This is a disadvantage that both phallometry and the IRAP have relative to the IAT, which has a comparable low attrition rate to the IRAP perhaps due to its lower complexity. For example, in a study that used both the IAT and the IRAP, the attrition rate was 17% vs 29%, respectively (Golijani-Moghaddam et al., 2013). The IRAP’s more stringent performance criteria may also have played a role; in the present and most other IRAP studies, participants were required to reach accuracy criterion of ≥ 80% correct responses and a median response latency of ≤ 2000 ms. In contrast, Snowden et al. (2020) discarded data from participants with ≤ 70% accuracy and applied no a priori response speed performance criteria (though outliers were scanned for). Future work should examine whether the sexual orientation IRAP and IAT have similar attrition rates when using similar performance criteria and whether results are impacted if the performance criteria for the IRAP are relaxed. That said, it is worth considering the implications for the present study: while there are no accepted standards for reasonable attrition rates in sex research, rates as high as these are considered threats to validity in randomized trials, and thus, the generalizability of these results may be limited.

Regarding sample size, it is notable that both samples of men and women were far larger than those included in most IRAP studies (Hussey, 2023b), and due to the extremely large point estimates of effect sizes in Timmins et al. (2016), it was expected that only a small sample size was required to reach reasonable power. While this appears to have been borne out in the present study, confidence intervals were wide, resulting in two issues: (1) the handful of non-significant results were unavoidably ambiguous, and (2) the precision of our effect size estimates was limited. Future research should replicate these results in even larger samples, which allow for precise estimates of effect sizes, as well as equivalence testing, as mentioned above (Lakens et al., 2018). The results of the current study will be extremely informative in planning this future work.

In conclusion, the results of the present study suggest that the sexual orientation IRAP has an ability to differentiate heterosexual, bisexual, and gay men from one another, and heterosexual women from lesbian/gay and bisexual women. These findings suggest the IRAP is a useful ex situ measure of sexual orientation and add to the existing scientific discussion concerning sexual orientation in women (Chivers, 2017).

## Supplementary Information

Below is the link to the electronic supplementary material.

Supplementary file1 (DOCX 18 KB)

## Data Availability

Available on request.

## Calculation of Sexual Orientation *D-*IRAP Scores

Individual response latency data were transformed into *D*-IRAP scores (see Barnes-Holmes et al., 2010a, 2010b) using an adaptation of the Greenwald et al. (2003) *D*-algorithm. Specifically, the process involved in the calculation of *D*-IRAP scores was as follows:

(i) only response latency data from test-blocks were used;
(ii) latencies above 10,000 ms from the dataset were eliminated;
(iii) all data for a participant were removed if they produced more than 10% of test-block trials with latencies less than 300 ms;
(iv) 12 standard deviations for the four trial types were computed: four for the response-latencies from test-blocks 1 and 2, four from the latencies from test-blocks 3 and 4, and a further four from test-blocks 5 and 6;
(v) 24 mean latencies for the four trial types in each test-block were calculated;
(vi) difference scores were calculated for each of the four trial types, for each pair of test blocks, by subtracting the mean latency of the male-attractive block from the mean latency of the corresponding female-attractive block;
(vii) each difference score was divided by its corresponding standard deviation from step 4, yielding 12 *D*-IRAP scores; one score for each trial-type for each pair of test blocks;
(viii) *D*-IRAP scores for a pair of blocks were removed if the participant dropped below the 80% correct responses rate or above the 2000 ms median response time in either block in the pair;
(ix) all data for a participant were discarded if the performance criteria are not met across two or three pairs of test blocks;
(x) four overall trial-type *D*-IRAP scores were calculated by averaging the three (or two) *D*-IRAP scores for each trial type across the test blocks;
(xi) two *D*-IRAP scores, one for male pictures and one for female pictures, were then calculated by averaging the two female picture trial-type scores and then the two male picture trial-type scores;
(xii) an overall *D*-IRAP score was calculated by averaging the male and female trial-type scores from the previous step.

Note that step (x) diverges with most previous work, but maximizes data retention and has been used previously, specifically with the Sexual Orientation IRAP (Timmins et al., 2016).

## Footnotes

1 And with the helpful suggestions of an anonymous reviewer.

2 For the purposes of this study, the term “gynesexual” will be used to refer to heterosexual men and lesbian/gay women collectively, and “androsexual” will be used to refer to gay men and heterosexual women collectively. This is in line with the utility provided by the synonyms “gynephilic” and “androphilic” (Bailey et al., 2016), but the suffix “-sexual” is retained to reflect the use of “-sexual” in sexual orientations and to avoid the negative connotations of “-philic.”.

## Code Availability

Available on request.
