# Supplementary material for: The Implicit Relational Assessment Procedure as a Measure of Sexual Orientation in Heterosexual, Bisexual, and Lesbian/Gay Men and Women
Source: Arch Sex Behav. 2025 Nov 10;54(10):4191–207. doi: 10.1007/s10508-025-03241-z (PMC12753572; doi:10.1007/s10508-025-03241-z)
Supplement: Supplementary file 1 — Supplementary file1 (DOCX 18 KB) [file 10508_2025_3241_MOESM1_ESM.docx]

| **Supplementary Materials Appendix B**  *Areas Under the Curve and 95% Confidence Intervals for IRAP Scores Comparing Gynesexual, Androsexual and Bisexual Women and Men classified on the basis of self-reported sexual attraction.* | | | | | | |
| --- | --- | --- | --- | --- | --- | --- |
|  | Women | | | | | |
|  | Overall D IRAP Score | | Female D IRAP Score | | Male D IRAP Score | |
| Variable | *AUC* [95% CI] | *p* | *AUC* [95% CI] | *p* | *AUC* [95% CI] | *p* |
| Gynesexual vs Androsexual | **.86 [.77, .96]** | **<.001** | **.92 [.85, .99]** | **<.001** | **.70 [.56, .84]** | **.006** |
| Gynesexual vs Bisexual | ***.70 [.58, .82]*** | ***.001*** | ***.73 [.61, .84]*** | ***<.001*** | .61 [.48, .74] | .097 |
| Bisexual vs Androsexual | **.73 [.60, .86]** | **<.001** | **.79 [.67, .90]** | **<.001** | *.60 [.46, .75]* | *.170* |
|  | Men | | | | | |
|  | Overall D IRAP Score | | Female D IRAP Score | | Male D IRAP Score | |
| Variable | *AUC* [95% CI] | *p* | *AUC* [95% CI] | *p* | *AUC* [95% CI] | *p* |
| Gynesexual vs Androsexual | **.92 [.86, .98]** | **<.001** | **.87 [.79, .95]** | **<.001** | **.84 [.74, .93]** | **<.001** |
| Gynesexual vs Bisexual | **.81 [.69, .91]** | **<.001** | **.75 [.63, .87]** | **.001** | **.74 [.62, .87]** | **<.001** |
| Bisexual vs Androsexual | **.72 [.60, .85]** | **.001** | **.66 [.52, .79]** | **.026** | **.67 [.54, .81]** | **.012** |
| *Note.* AUC = Area Under the Curve, Bolded = Significant (α = 0.05), Italics = Significance/non-significance differs from primary analysis. | | | | | | |
